# Some Clinical Applications of the Male and Female Sex Hormones
*A paper read before the Bristol Medico-Chirurgical Society on Wednesday, February 9th, 1938.


**Published:** 1938

**Authors:** G. L. Foss


### SOME CLINICAL APPLICATIONS OF THE MALE AND FEMALE SEX HORMONES.*

BY

G. L. Foss, M.B., B.Ch.

---

The purpose of this communication is to give some idea of the work done during the past three and a half years, and at the same time to afford a general impression of some of the uses to which the male and female hormones can be applied.

This subject has developed very rapidly in the last seven years. Owing to advances in bio-chemistry, clinicians have active standardized preparations of both hormones readily available.

### The Female Hormone.

There are two ovarian hormones, œstradiol and progesterone. Œstradiol benzoate is an ester of the most powerful of the œstrogens, which is identical with that of the follicular hormone in the ovaries. For clinical use it is available in an oily solution in two doses: 1 mgm. or 10,000 I.B.U.† and 5 mgm. or 50,000 I.B.U. In this form it is slowly absorbed and is of lasting action, having an even and continued effect over a period of about four days, thus obviating the need of daily intra-muscular injections.

* A paper read before the Bristol Medico-Chirurgical Society on Wednesday, February 9th, 1938.

† International Benzoate Unit.

The route of administration of this hormone is rather important. For a prolonged effect intramuscular injections are given of this oil solution of œstradiol benzoate. For a slight but rapid effect, œstrone is administered in tablet form by mouth at frequent intervals, as both assimilation and excretion are very rapid. As a local application which is of considerable help in some cases, it may be prescribed as a cream or vaginal suppository for women and for small children in the form of vaginal capsules.

Progesterone, the hormone of the corpus luteum, is only active following preliminary action of adequate œstradiol and as my experiences are confined almost entirely to the follicular hormone, I will mention progesterone only briefly.

Before one can readily employ this therapy, its physiological actions must be understood. The use of œstradiol benzoate depends on two factors: (1) A substitution or supplement of the normal follicular hormone, and (2) A stimulation to the anterior pituitary.

First I will try to explain the normal processes of the human sexual cycle.*

The gonadotrophic fraction of the secretion of the anterior pituitary or master gland stimulates maturation of the follicle and production of œstradiol from the granulosa and theca cells. Ovulation occurs about the fourteenth day and is followed by corpus luteum formation. This process takes place in cyclical rhythm about every twenty-eight days throughout sexual life. As a result of such stimulation, the two hormones of

* A film produced by Professor Loeser and kindly lent by Messrs. Schering, Limited, was projected to illustrate the normal process of menstruation.

the ovary are elaborated—œstradiol in the follicle and progesterone and œstradiol from the corpus luteum.

The continuous supply of these hormones in the blood stream has a specific action on the endometrium. During the first fourteen days following cessation of the previous uterine bleeding, a proliferation or follicular phase takes place in the resting glands in the basal layer of endometrium, which alone remains after menstruation. From this basal layer are built up long winding glands with cylindrical epithelium. This tissue increases its thickness altogether seven times and the ratio of gland tissue to stroma increases.

At about the fourteenth day, the follicle becomes mature and owing to increased internal pressure, it bursts and the ovum is extruded. Now organization takes place in the hæmorrhagic follicle and a corpus luteum is formed, and under the action of its progesterone, a new change is seen in the endometrium. The columnar cells of the glandular tissue take on a secretory function. Granules of glycogen appear in the cytoplasm and the nuclei are pushed towards the basal ends of the cells. Finally, at about the twenty-fourth day of the cycle, these cells pour their glycogen content into the lumina of the glands which are distended and dilated. The increased pressure in these glands causes them to become tortuous and to take on a typical cork-screw appearance. The glandular element compresses the cytogenic stroma and becomes more evident.

Parallel with this glandular change in the proliferation and secretory phases there is also marked change in the stroma cells, which rapidly enlarge, and this tissue becomes very congested and vascular. Towards the twenty-fourth day of the cycle, these cells become œdematous and rather like decidua cells ; leucocytic infiltration is found in the region of the vessels, and after death of the corpus luteum, intercellular hæmorrhages take place. Finally, on disintegration the whole of this rich congested endometrium is shed, being visible evidence of this cyclical function.

In summary, the follicle elaborates œstradiol which induces the proliferation phase in the endometrium and the corpus luteum elaborates progesterone which converts this proliferation phase into a secretory phase, suitable for nidation of a fertilized ovum.

Menstrual bleeding may take place from a luteal or secretory phase in the normal way, or again, it may occur from a proliferation or follicular phase only, when it is spoken of as "anovular bleeding." Œstradiol is produced by the follicle and also by the corpus luteum in addition to progesterone and its concentration does not drop until the last few days of the cycle. Bleeding in absence of ovulation occurs only following diminution of this concentration below the threshold value ; which is that amount of œstradiol normally necessary to bring the endometrium to that stage of proliferation in fourteen days, which will be followed by uterine bleeding on withdrawal.

The modern opinion is that the luteal or secretory phase is constant and lasts fourteen days, whereas the follicular or proliferation phase is variable, depending on the rate of maturation of the follicle and the time of ovulation. Both maturation of the follicle and ovulation are necessary antecedents to the formation of the normal corpus luteum. Thus the luteal phase is indicative of the occurrence of previous ovulation.

Whenever therapy is given in an endeavour to re-establish normal menstrual rhythm during sexual life, it must imitate the proliferation phase as nearly as possible. Menstruation is a mere physical expression of this pituitary-ovarian rhythm, and therefore one's aim in using œstradiol benzoate is to restore this cycle, in the hope that by a process of stimulation and substitution this may be continued and the reflex established.

At puberty, follicular maturation begins and under the rhythmic action of œstradiol the girl becomes a woman. The breasts increase in size and the genitalia attain the adult form. The infantile uterus grows and the myometrium develops. From now on until the menopause, the follicular hormone controls the uterus.

In addition to the changes in the endometrium, the tone of the myometrium also is maintained by the balance of follicular and luteal hormones. Œstradiol sensitizes it to the action of pitocin, whereas progesterone de-sensitizes it.

During pregnancy the follicular hormone in the circulation is much increased. It is manufactured by the placenta and causes growth of the uterus to protect the fœtus, whereas progesterone de-sensitizes it and prevents contraction. At term, the activity of the œstrogen changes and sensitizes the myometrium to pitocin, thus setting up contractions.

A healthy condition of the vagina and vulva is also assured by œstradiol. It increases the vascularity of the deeper layers of the dermis while it causes a proliferation-cornification of the squamous epithelium. The vaginal smear test of Papanicolaou[13] has demonstrated a cyclical alteration in these cells parallel with the secretion of follicular hormone.

The secretion of glycogen in these cells is maintained also, so that when they break down lactic acid is formed under the action of Döderlein's bacilli, and a low p H. is found in the normal vagina which prevents growth of pathogenic organisms.

The stimulation of the pituitary by small doses of œstradiol has been mentioned already. As so often occurs with the endocrine glands, this hormone has a diphasic action : large doses inhibit the pituitary. Normally there is a delicate balance between the pituitary and ovary, so that as one increases in action the other is inhibited, and this is probably materially responsible for the cyclical rhythm.

On termination of ovarian activity and disappearance of œstradiol from the circulation the secondary sex organs atrophy and the menopause occurs. Immediately it is found that the output of the gonadotropic factor is increased owing to removal of ovarian inhibition, and in the menopause there is prolanuria. The upheaval which takes place in the endocrine system at this time is the cause of the distressing symptoms which are so common until readjustment occurs.

---

After this brief survey of the main facts about the female hormone, I will endeavour to explain some of the applications for which I have used it. This may no longer be the correct treatment when synthetic prolan A. and B. are available.

### THERAPY.

1. *Premature Babies.* I have no personal experience in this field. Dr. Potter has treated a number of babies at the Maternity Hospital and so has Dr. Phillips at Southmead. I believe it is the routine treatment at both hospitals and is said to diminish anxiety. So far, no deaths of premature babies have occurred since it was started at the Maternity Hospital, in Dr. Potter's series. It is given by mouth in the feeds.

2. *Vulvo-vaginitis in Small Children.* Opinions seem to differ on this use of œstradiol. As far back as 1933, Lewis[11], Brown[1], Miller[12], and others in America gave daily intra-muscular injections and claimed good results. The modern application, however, which avoids any general stimulation of premature puberty, consists of local application of gelatine-covered capsules inserted into the vagina. Œstrone tablets may be given by mouth as well. My results with six cases, four of which were definitely gonococcal, have been promising, and after a month smears and cultures were negative. Two such cases had been treated for four to five months previously with routine treatment and were still positive.

3. *Menstrual Irregularities.* Before treating any case of menstrual irregularity, a careful history should be obtained, and any organic disease excluded by a complete medical and gynæcological examination. Preferably, in cases which still have some menstruation, patients should have a diagnostic curettage just prior to, or on the first day of bleeding, to see whether ovulation takes place.

In all cases one must remember that the object is to restore the normal function and the proliferative phase must be built up by doses given on the first, fourth, eighth, eleventh and fifteenth days of the cycle. In this way, according to the nature of the œstrin deficiency, either supplementary and stimulatory doses of 1 mgm. or substitution doses of 5 mgm. are given. It is hoped that by so doing, either ovulation may be stimulated and the normal secretory phase will follow, or the follicular phase will be built up to a sufficient level, so that the naturally-occurring luteal hormone may have its effect.

*Oligomenorrhœa* or infrequent menstruation, *Hypomenorrhœa* or scanty menstruation, and *Epimenorrhœa* or too frequent menstruation, are all treated on the same lines in cyclical rhythm. The dosage, of course, varies according to the severity or duration and nature of the individual disorder. Treatment is necessary probably for four to twelve months and dosage should be reduced only gradually, always injecting the largest dose on the fifteenth day of the cycle. The prognosis is best for cases of oligomenorrhœa as long as this is not associated with menorrhagia, when œstradiol therapy alone is useless. Cases of hypomenorrhœa have usually an increased loss during treatment, but this tends to relapse after.

*Amenorrhœa* needs slightly different treatment, as here there is no cycle at all. Menstruation in mild cases of secondary amenorrhœa, which are probably subliminal in type, can usually be started after five injections of 5 mgm. on the first, fourth, eighth, eleventh, and fifteenth days. After about seven days' rest, bleeding begins. As soon as bleeding has finished, cyclical rhythm is continued by injections in the proliferative phase as before.

Cases of longer standing with uterine hypoplasia are best treated with bi-weekly injections for one to two months to develop the whole uterus. Bleeding will probably start on ceasing injections. Immediately this has finished, cyclical dosage is continued for six to twelve months. Failure to induce the onset of bleeding after such treatment should make one suspect pregnancy and an Ascheim-Zondek test should be carried out before diagnostic curettage is attempted. Often a brief course of treatment is sufficient to aid fertilization and nidation. After gradual reduction of dosage by injection, oral therapy can be substituted for a few more months. By this means, as I have proved on several occasions, true menstruation from a secretory-phase endometrium following ovulation can be re-established, using œstradiol therapy alone. Spontaneous menstruation after cessation of treatment is probably only to be expected in from 25 to 40 per cent. of cases and time will possibly show a tendency to relapse. There may be a large pituitary, hypothalamic or even cerebral factor responsible in these cases.

*Primary Amenorrhœa.* I have treated two cases of this rare condition. The first, a girl of 20 years, failed to menstruate owing to my dosage being too small. The second was a girl of 25 years[8] who had never menstruated. I built up her uterus for four months with a total dose of 430 mgm. and after a rest of six days gave five daily injections of 5 mgm. progesterone, and two days later she saw her first period. Since then during the last eight months, she has had seven periods with œstradiol benzoate only.

*Primary Sterility in the Female.* I have treated only about six cases, all of which were associated with some menstrual irregularity and genital hypoplasia.

All were treated with œstradiol benzoate on similar lines to that already stated: two have successfully produced babies, and one is pregnant.

*Action on the Myometrium.* No amount of œstradiol benzoate will affect an established pregnancy, as its action is antagonized by progesterone ; but for intra-uterine death, where there is not sufficient œstrin in circulation to sensitize the myometrium to pitocin, 1 to 5 mgm. of œstradiol benzoate should be given every eight hours up to eight days and then medical induction is successful in 80 per cent. of cases. According to Jeffcoate[10] some help may be obtained in primary uterine inertia by 2 mgm. hourly for ten injections.

*True Dysmenorrhœa,* or spasm of the muscle, occurs once bleeding starts and is now thought to be due to lack of sufficient progesterone. I have found that œstradiol benzoate, given in cyclical rhythm for four to twelve months, is sufficient to remedy this. The course may have to be repeated. Nearly all my cases were associated with uterine hypoplasia and some form of irregular or scanty bleeding. Invariably if the loss were increased the pain diminished. Probably the extra dose of œstrin enabled full luteinization to occur, thus controlling the spasm of the muscle. Results are lasting in a large number of cases.

*Menorrhalgia,* or congestive or extrinsic pain, is also benefited. The type of case with menorrhagia, however, is not relieved by this therapy. The neurotic type also is disappointing.

*Action on the Breast.* Practically all cases treated for any length of time show some mammary development or stimulation, from slight tenderness or soreness around the nipple to a considerable enlargement.

This may be a useful application for those who desire a fuller bust. During lactation the supply of milk can be dried up, as œstradiol and œstrone concentrate the protein in the milk, as was shown experimentally by Folley[4] on the cow. Dr. Phillips, I believe, is finding œstrone by mouth useful for this purpose. So far, I have treated only six cases. Dosage is usually 2,000 I.B.U. two-hourly by mouth for about four to eight days, with only fluid restriction. Occasionally injections of 1 to 5 mgm. twice daily may be necessary at first, if there is a lot of milk.

*The Control of Menstruation.* By the simple measure of prolonging the proliferation phase and maintaining a super-threshold dose of œstradiol benzoate until the expected date of onset has passed, menstruation may be postponed[6] for a few days or as long as desired. The simplest way of doing this is by giving 5 mgm. every three days, starting about the tenth day of the cycle. Ovulation is thus inhibited. Larger and more frequent doses are required if the patient is not seen till after the thirteenth day.

Early induction of bleeding can also be obtained by hastening an early proliferation phase and then converting it into a definite secretory phase by progesterone: bleeding will start prematurely on withdrawal. It is wise not to repeat this application too often and it should only be used to avoid such occasions as a wedding day or tennis championship.

*The Menopause.* Most of the symptoms of the climacteric, including hot flushes and even endocrine arthritis, have been controlled by the smaller or moderate doses given by mouth or by injection. Some indication of the basic level required is given by the vaginal smear test of Papanicolaou.[13] The menopause is one of the best known and most successful indications for œstrin therapy.

*Vulvo-vaginitis,* including idiopathic pruritus vulvæ, kraurosis, leukoplakia and atrophic senile vaginitis make up forty cases in my series.[5] By no means are they all post-menopausal and some of the worst have been menstruating regularly. Brian Swift[15] discovered their association with achlorhydria and maintains that this is responsible for a vitamin A deficiency. In the absence of this protective factor, infection or trauma soon set up chronic irritation in the delicate vulvo-vaginal epithelium, especially after the menopause, when this epithelium is thin and atrophic. In my series, 37 per cent. had total and 37 per cent. partial achlorhydria. The treatment then consists of a high vitamin diet, hydrochloric acid by mouth with halibut or cod liver oil. Œstradiol benzoate is given in doses varying from 5 to 25 mgm., bi-weekly at first. Relief is obtained in one to two months, when dosage can be cautiously reduced. A recent advance is the local application of œstradiol in vaginal suppositories or in a cream, thus reducing the necessity for the larger doses.

As this is entirely substitution therapy, treatment is likely to be prolonged, but with local treatment small weekly injections are often adequate. After large doses, even in old women of 80 years, withdrawal bleeding is usual and its probable occurrence should be explained.

*Inhibition of Pituitary Function.* A case of acromegaly treated with 1,215 mgm. for seven months has only shown how apparently innocuous large doses are, but her condition is stationary. Pituitary basophilism (Cushing's Disease) has been treated also in America by Dunn.[3] I have treated a woman of 70 years with 1,600 mgm. in eight months. She had a fungating scirrhus carcinoma of the breast which was painful and there was a large ulcer with a foul-smelling discharge. The only effect that I found was that the ulcer attempted to heal and the discharge to lessen. Pain was reduced and the smell disappeared. This should allay any fears of cancer stimulation by this therapy. Œstradiol benzoate has been tried with varying success in some other conditions.

### THE MALE HORMONE.

This is available at present as testosterone propionate. This ester dissolved in oily solution is found to be most potent and the effect lasts about seven to fourteen days. The uses of the male hormone are indefinite at present, partly because its effect is not completely known and partly because the human dosage has not been fully decided. It is said to stimulate the growth and activity of the secondary sex organs such as the penis, prostate, Cowper's glands, vesiculæ seminales, etc., and, in conjunction with the supra-renal cortex, is responsible probably for the male psyche.

Recently I have had the opportunity of investigating its effect on a post-puberal eunuch of 38 years, who was wounded in the War.[7] His penis was unharmed but the lacerated remains of his testicles were removed by Mr. Stuart Stock in 1918. This patient married in 1923, and was impotent until 1937, when I was able to restore his sexual vigour. In a week, after daily injection of 20 mgm. of testosterone propionate, he developed painful priapism. Normal coitus was possible now for the first time and full erotic sensation and orgasm were obtained, but without any ejaculation. This normal condition was maintained by a weekly dose of 40 mgm. Coitus took place with satisfaction to both parties and libido was excessive. During twelve weeks of treatment his weight increased by 16 lb. and his collars became too small, but in the nine weeks after treatment ceased he lost 10 lb. As this patient co-operates so well, I am now continuing therapy with a view to elucidating other points of interest. Hamilton,[9] in America, has published recently a case of hypo-gonadism in a medical student who re-acted similarly to testosterone propionate.

From these human experiments some idea of the human dosage can be obtained; although in normal man another factor has to be considered, namely the antagonistic effect of female hormone. Œstrin occurs in the male gonad in large quantities and is excreted in male urine. The richest source of œstrin yet discovered is the testicle of the stallion, which Zondek[16] has estimated as containing 500 times more œstrin than the ovary of a sexually mature mare. Œstrin is derived probably from testosterone by a progressive degeneration of this sterol.

The bi-sexual output of hormones from the testicles has given rise to the theory of prostatic hypertrophy which is associated with the male climacteric. Zuckerman's[21] view after considerable research with dogs and monkeys is that there is an imbalance between these two hormones in aged animals and

œstrin, which normally is inhibited by testosterone, now becomes dominant in action. The prostate of a newly-born baby is said to become smaller after birth owing to the removal of the influence of the maternal œstrin.

It has been shown that the prostate of dogs when stimulated by œstrin administration gives an identical microscopic picture to that of normal adenomatous hypertrophy; and Zuckerman[17] thinks that the uterus masculinus in man under the action of unopposed œstrin in later life undergoes cystic hyperplasia such as that which takes place in the female endometrium. The hypertrophied middle lobe is thought by some to compress the true prostatic tissue, which after surgical removal of the adenoma is left behind as a capsule in which lie the ejaculatory ducts. Further, Zuckerman[18] has shown that testosterone propionate will inhibit the effects of œstrone on the prostate of the immature Rhesus monkey, if given in the ratio of 7 to 1.

As soon as this hormone was available in adequate dosage and sufficient quantity for clinical trial, I started to treat a series of cases with prostatic symptoms—senility, impotence, etc. The enthusiasm which the results of animal research led one to adopt has been damped considerably after using this therapy for the past year. My first difficulty was to obtain suitable cases. This may sound incredible until it is realized that in hospital practice few patients are sent in unless they are on the threshold of retention and in poor general condition. When perhaps they are suitable for a preliminary attempt at hormone therapy, the keen surgeon immediately seizes them and shells out the offending gland.

The next difficulty was to find patients in my own practice with symptoms still in an early stage. Every man above 50 years who came up complaining of a toothache or other minor ailment was questioned in detail about his uro-genital function and submitted to a rectal examination. By this means I have collected a small series of eighteen cases during a period of twelve months.

These may be grouped into (*a*) Advanced prostatic hypertrophy, and (*b*) Early cases with only moderate enlargement.

For the late cases where there is much adenomatous hypertrophy and considerable fibrosis, one cannot hope to achieve very much in reduction of the size of the gland, but the improvement which occurs in urinary symptoms is remarkable. There is some reduction in the nocturnal frequency after a week or so. In some, the stream is improved and urgency is lessened and they can hold their water better. There is not such a marked change in diurnal frequency. This effect is thought to be due to the increased tone of the bladder wall.

My first cases were given weekly injections of 40 mgm. (a dose recommended by Zuckerman and Greene[20]), but very soon I found that improvement was more rapid if injections were given daily in doses of 40 to 50 mgm. In one case of a man of 70 years the prostate was as large as a small tangerine and this patient got out of bed three or four times a night. With daily injections of high dosage this frequency was reduced steadily and he seemed to be progressing favourably until he had an acute attack of diarrhœa. The extra congestion was just sufficient to complete the obstruction and he developed retention. Mr. Jackman kindly carried out a two-stage prostatectomy and he shelled out a large prostate weighing 85 grams. Section of this revealed no change attributable to the male hormone, but this could scarcely be expected after only eighteen days' treatment.

Male hormone therapy on experimental grounds was promising, but the clinical results have been only partially successful. However, there is a definite improvement physically in most patients treated, erections are often more ready and powerful, even in aged bachelors, and they describe a feeling of well-being. Nocturnal frequency is reduced in nearly all cases and in some to nothing. The stream improves and there is less urgency and delay. So far, I have been able to detect diminution in size of only one prostate which has had a year's treatment.

As regards other features of clinical improvement, the passage of catheters for measuring the volume of residual urine before and after treatment is not ideal and is difficult in general practice.

I think treatment with testosterone propionate should be maintained for a year or two, giving about 40 mgms. every fortnight. A small dose of œstradiol benzoate, 0.05 mgm., is given now with each dose of testosterone propionate, as this was found by Steinach and Kun[14] to increase its action. The latest suggestion by Cramer and Horning[2] for the treatment of prostatic enlargement consists of giving the pituitary thyrotropic hormone, which is antagonistic to œstradiol, and I hope to start clinical trials shortly in five cases.

Results up to the present suggest that if treatment is given in adequate dosage for a long period, early cases may well escape a future retention and prostatectomy, but it must be remembered that surgical intervention is the only treatment of prostatic obstruction. Vas ligation or radiation of the testicle by X-rays in conjunction with such endocrine treatment may possibly become the future technique. At least five years must elapse before a definite opinion may be formed of the final value of this therapy.

Testosterone propionate is useful in certain cases of impotence and sexual asthenia and has a tonic effect on the male similar to that of œstradiol benzoate on the female. It is useful for early senility and previously weak, semi-flaccid erections are strengthened and libido returns. Mental activity and bodily well-being is increased.

### ACTION ON THE FEMALE.

In the normal mature female Rhesus monkey testosterone propionate has been shown experimentally by Zuckerman[19] to inhibit ovulation, follicular growth and luteinization. This may be developed into a method for securing temporary sterility in the human female.

In some of my cases, if dosage were continued throughout a cycle, the endometrium was shown by curettage to remain in the resting stage. This action suggests a method of treatment for menorrhagia and other benign uterine hæmorrhage. However, I have found its action to be erratic, although this may be due to incomplete diagnosis or incorrect dosage. Male hormone is very useful in some cases, and I quote the following example. A patient aged 45 years with severe menorrhagia of the early menopause had been treated with drugs and curettage, but bleeding continued and was due to metropathia hæmorrhagica. Her condition was so bad and she was so anæmic that hysterectomy was proposed. Meanwhile testosterone propionate was given twice weekly following cessation of one copious bleeding. Forty mgm. were injected on the fifth, ninth, twelfth, seventeenth and twentieth days after this. She was curetted on the twenty-fifth day and the endometrium was in the resting stage: scanty bleeding followed three days later. Immediately five more injections were given every four days and she was curetted on the twentieth day, when the endometrium once more was found to be in the resting stage. Seven days later I failed to remove any endometrium with the biopsy curette. No more bleeding was seen for twenty-one weeks, when this was normal.

Further trials for this condition are being made. Experiences with such animal and human therapy show that there is no ill effect, except perhaps slight enlargement of the clitoris.

My thanks are due to Mr. Shepherd, Dr. Lily Baker, and Dr. Potter for their help and co-operation and for allowing me to treat their cases in the Gynæcological Out-patient Department, and to Dr. Fraser for the sections and pathological reports: also to Professor Davie for facilities to obtain slides and photomicrographs. Samples of Progynon and Testoviron have been generously supplied by Messrs. Schering, Limited.

### REFERENCES.

[1] Brown, J., *Jour. Amer. Med. Assoc.*, 1934, cii. 1,293.
[2] Cramer, W., and Horning, E. S., *Lancet*, 1938, i. 72.
[3] Dunn, C. W., *Lancet*, 1937, ii. 344; *Ibid.*, 1937, ii. 828.

[4] Folley, S. J., *Biochem. Jour.*, 1936, 30, ii. 2,262.

[5] Foss, G. L., *Jour. Obst. and Gynæc. Brit. Emp.*, 1936, xliii. 1,091.

[6] Foss, G. L., *Brit. Med. Jour.*, 1937, ii. 10.

[7] Foss, G. L., *Lancet*, 1937, ii. 1,307.

[8] Foss, G. L., *Jour. Obst. and Gynæc. Brit. Emp.*, 1938, xlv. 74.

[9] Hamilton, J. B., *Endocrinology*, 1937, xxi. 649.

[10] Jeffcoate, T. N. A., *Brit. Med. Jour.*, 1937, i. 674, 720.

[11] Lewis, R. M., *Amer. Jour. Obst. and Gynec.*, 1933, xxvi. 593.

[12] Miller, J. R., *Amer. Jour. Obst. and Gynec.*, 1935, xxix. 553.

[13] Papanicolaou, G. N., and Shorr, E., *Amer. Jour. Obst. and Gynec.*, 1936, xxxi. 806.

[14] Steinach, E., and Kun, H., *Lancet*, 1937, ii. 845.

[15] Swift, B. H., *Jour. Obst. and Gynæc. Brit. Emp.*, 1936, xliii. 1,053.

[16] Zondek, H., *Diseases of Endocrine Glands*, London, 1935.

[17] Zuckerman, S., *Proc. Roy. Soc. Med.*, 1936, xxix. 1,557.

[18] Zuckerman, S., *Lancet*, 1936, ii. 1,259.

[19] Zuckerman, S., *Lancet*, 1937, ii. 676.

[20] Zuckerman, S., and Greene, R., *Lancet*, 1936, ii. 1,433.

[21] Zuckerman, S., and Groome, J. R., *Jour. Path. and Bact.*, 1937, xliv. 113.

